# A new flagellated dispersion stage in *Paraphysoderma sedebokerense*, a pathogen of *Haematococcus pluvialis*

**DOI:** 10.1007/s10811-015-0700-8

**Published:** 2015-10-18

**Authors:** Martina Strittmatter, Tiago Guerra, Joana Silva, Claire M. M. Gachon

**Affiliations:** Scottish Marine Institute, Scottish Association for Marine Science, Oban, Argyll PA37 1QA UK; A4F—Algae For Future SA, Campus do Lumiar—Edifício E—R/C, Estrada do Paço do Lumiar, 1648-038 Lisbon, Portugal

**Keywords:** Pathogen, Green alga, Blastocladiales, Zoospores, Amoebae, Flagellum, Life cycle

## Abstract

The blastocladialean fungus *Paraphysoderma sedebokerense* Boussiba, Zarka and James is a devastating pathogen of the commercially valuable green microalga *Haematococcus pluvialis*, a natural source of the carotenoid pigment astaxanthin. First identified in commercial *Haematococcus* cultivation facilities, *P. sedebokerense* is hypothesised to have a complex life cycle that switches between a vegetative and a resting phase depending on favourable or unfavourable growth conditions. Rather unusually for blastocladialean fungi, *P. sedebokerense* was described as lacking flagellated zoospores and only propagating via aplanosporic amoeboid cells. However, during repeated microscopic observation of *P. sedebokerense* cultivated in optimal conditions, we detected fast-swimming, transiently uniflagellated zoospores which rapidly transform into infectious amoeboid swarmers, the existence of which suggests a closer than previously thought relatedness of *P. sedebokerense* to its sister genera *Physoderma* and *Urophlyctis*. Additionally, we found some morphological and physiological differences between amoeboid swarmers and discuss hypotheses about their significance. These amoeboid and flagellated propagules are key to the dissemination of *P. sedebokerense* and are probably also the life stages most vulnerable to adverse environmental conditions. They are therefore a prime target for the development of disease management protocols in industrial cultivation facilities, a goal which requires a detailed understanding of their physiology.

**Electronic supplementary material:**

The online version of this article (doi:10.1007/s10811-015-0700-8) contains supplementary material, which is available to authorized users.

## Introduction

*Haematococcus pluvialis* Flotow is a chlorophycean freshwater microalga that attracts increasing interest for industrial mass-scale cultivation. Under stressful conditions, for instance nitrogen depletion, this species produces high levels of the ketocarotenoid astaxanthin (Droop 1955; Boussiba 2000). *H. pluvialis* therefore represents an important natural source of this pigment (Lorenz and Cysewski 2000) which is frequently used in cosmetics, in nutraceuticals and as animal feed. A corollary of increasing algal commercial cultivation at industrial-scale levels is an ever greater interest in the pathogens that hamper production (Carney and Lane 2014). Although some green algal pathogens (e.g. *Chlorella* virus) have been described in natural systems for a long time, mass cultivation in recent years has accelerated the rate of disease discovery (Shin et al. 2001; Letcher et al. 2013; Carney et al. 2014; Chen et al. 2014). Amongst those, Hoffman et al. (2008) first reported *Paraphysoderma sedebokerense* as a new pathogen of *H. pluvialis* in Israel and established its taxonomic position in the *Blastocladiomycota* closely related to the plant pathogen genus *Physoderma* (Hoffman et al. 2008; James et al. 2012). Since then, patent applications relating to its control have been developed in the USA and China, strongly suggesting that *P. sedebokerense* causes widespread production losses in industrial facilities across the world (McBride et al. 2013; Zhang et al. 2013).

**Fig. 1:**
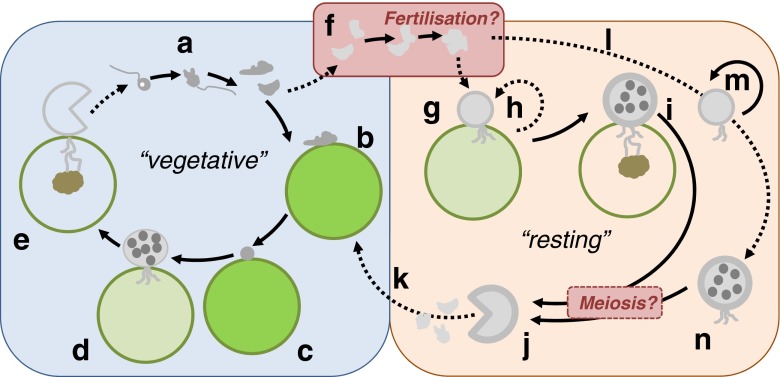
An updated life cycle for *P. sedebokerense* (*a*–*e*). Vegetative, putative haploid (epibiotic) phase of the life cycle: posteriorly uniflagellated zoospores (*a*) transition into infectious amoebae (*b*) that encyst at the surface of a host *Haematococcus* cell (*c*). A rhizoid is formed inside the algal host and sustains the differentiation of a thin-walled sporangium (*d*) that ultimately releases a new generation of swarmers (*e*). A fertilisation event between two amoebae might lead to transition into the resting phase of the life cycle (*f*). *g*–*h* Resting, putative diploid phase of the life cycle (as per Hoffman et al. 2008): in unfavourable growth conditions, the differentiating sporangium (*g*) originating from the encystment of a diploid amoeba can either undergo asexual reproduction (*h*) or evolve a thick wall and form a resting sporangium, still resulting into the death of the host cell (*i*). Diploid amoebae can also grow saprophytically, e.g. on agar medium (*l*–*n*) either undergoing asexual reproduction (*m*) or forming a resting sporangium (*n*) similar to *i*. By analogy with the closely related genera *Physoderma* and *Urophlyctis*, it is tantalising to suggest that meiosis occurs in the resting sporangia (*i*, *n*) giving rise to haploid swarmers (*j*). Transition between the vegetative (*f*) and resting (*k*) cycles would be triggered by fertilisation and meiosis, respectively. In contrast to the vegetative cycle of *P. sedebokerense*, Hoffman et al. showed that the amoeboid swarmers released from the resting sporangium (*j*) are devoid of flagella. *Full and dotted arrows* represent established and hypothesised life cycle transitions

Hoffman et al. (2008) also identified major steps in its complex life cycle (Fig. 1): Under favourable conditions, amoeboid swarmer cells settle, attach and encyst on the *Haematococcus* host cells. The encysted cell develops a germ tube which pierces the host cell wall and then forms a rhizoid system extending into the host cytoplasm. During the course of infection, the cyst develops into a vegetative sporangium. Hoffman and colleagues have postulated that amoeboid swarmers are released from vegetative sporangia which are able to move via pseudopodia. Under unfavourable conditions, the pathogen displays a second type of life cycle and forms a different type of sporangium, the so-called resting sporangium which can be distinguished by a thicker cell wall and dark opaque appearance. These resting sporangia can be found on the surface of its algal host but can also be grown saprophytically on nutrient agar. Like for the vegetative sporangium, amoeboid swarmer cells are released. Having failed to observe flagella on these swarmer cells, the authors concluded that *P. sedebokerense* is aplanosporic whereas its sister taxa *Physoderma* and *Urophlyctis* contain posteriorly uniflagellated zoospores (Porter et al. 2011). Our observations of *P. sedebokerense* in a laboratory-controlled pathosystem reveal the existence of transiently uniflagellated amoeboid zoospores and enable us to propose an updated, yet still partly hypothetical, life cycle for this species.

## Material and methods

### Culture conditions

*Haematococcus pluvialis* strain SCCAP K-0084 was cultivated in Bold Basal Medium with 3-fold Nitrogen and Vitamins (3N-BBM + V; CCAP 2015) at 25 °C and a light intensity of 80 μmol photons m^−2^ s^−1^ (12-h:12-h light–dark period) in 50-mL tissue culture flasks. A clonal isolate (Haem c1) was established by picking single cells spread on 3N-BBM + V agar medium and used as a host for propagating *Paraphysoderma sedebokerense* strain PS1 which was isolated from outdoor *H. pluvialis* cultures in Portugal. Subcultivation was routinely performed every 10–14 days by inoculating 2–5 mL of *Haematococcus* culture into 15–18 mL of fresh 3N-BBM + V medium and adding 100–200 μL of *Paraphysoderma*-infected *Haematococcus* culture from the previous inoculation. The progress of infection and absence of eukaryotic contaminants in the culture were monitored on a biweekly basis for over a year. *P. sedebokerense* was also grown on chytrid growth medium agar as described by Hoffman et al. (2008) with the exception that 3N-BBM + V was used instead of mBG11. Cultures of infected *H. pluvialis* were spread on agar plates and incubated as mentioned above. After 6–8 weeks, prominent colonies of *P. sedebokerense* were visible which could be picked and transferred to new agar plates, resulting in host-free cultures of the pathogen. All biological material is available from the authors for non-commercial purposes upon request.

### Molecular identification

The DNA of infected *Haematococcus* material was extracted as previously described (Gachon et al. 2009), and PCR was performed as follows: 0.4 mM of each primer (MH2 and MH4) (Vandenkoornhuyse and Leyval 1998), 1× Taq PCR Master Mix (Qiagen), and 5 ng μL^−1^ DNA. Amplification was done with an initial denaturation step at 94 °C for 1 min followed by 35 cycles of denaturation at 94 °C for 1 min, annealing at 48 °C for 30 s, and extension at 72 °C for 2 min, followed by a final extension step at 72 °C for 10 min. The PCR products were then purified using the Qiaquick PCR purification kit (Qiagen) and sequenced using the two primers MH2 and MH4 (GATC LIGHTrun). The sequence has been deposited in GenBank under the accession number KT270356.

### Microscopy

Zoospore suspensions were produced by concentrating 2 mL of liquid *Paraphysoderma*-infected *Haematococcus* culture by centrifugation at 15,700×*g* for 5 min at room temperature. The pellet was then resuspended in 30 μL of culture medium and mounted on a microscope slide. Care was taken to properly wash down the outer-facing wall of the reaction tube in order to maximise the cell numbers. Observation was by differential interference contrast microscopy and pictures were recorded using an Axiocam HRc (Zeiss). In order to reveal lipid bodies in zoospore and amoeboid life stages, cultures were stained with Nile red (Greenspan et al. 1985). Cultures were concentrated by centrifugation and resuspended in 100 μL 3N-BBM + V medium and Nile red added to a final concentration of 40 μg mL^−1^ from a stock solution of 4 mg mL^−1^ dissolved in acetone. Calcofluor white was used to stain pathogen cell walls and was used at a final concentration of 2.5 μg mL^−1^. In both instances, labelling was performed for 10–15 min at room temperature in the dark. Cells were then pelleted by centrifugation as described above, resuspended in 30 μL 3N-BBM + V medium, mounted on a microscope slide, and observed by fluorescence microscopy (Nile red: Zeiss filter set 20, excitation BP 546/12, beam splitter FT 560, emission BP 575–640; Calcofluor white: Zeiss filter set 02, excitation G365, beam splitter FT395, emission LP420).

## Results and discussion

**Fig. 2:**
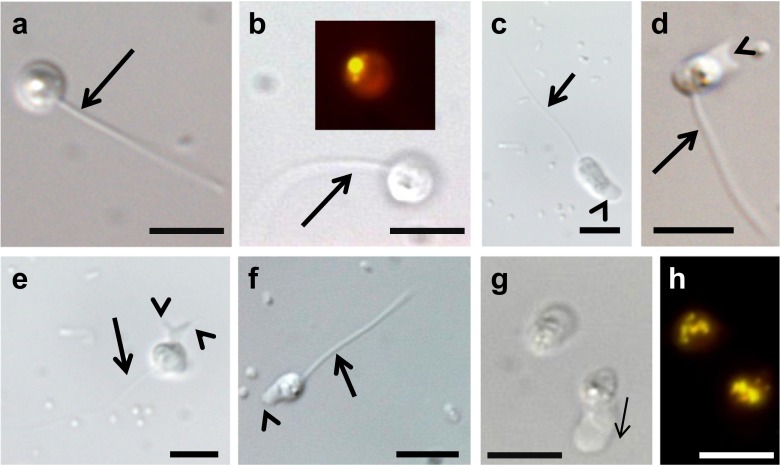
Transformation event of uniflagellated zoospores into the amoebal life stage of *P. sedebokerense* (**a**–**b**). Uniflagellated zoospores at the beginning of microscopic observations. *Inset* in **b** shows the lipid globule of the zoospore stained in Nile red. **c**–**f** Transition from a flagellated zoospore into the amoebal stage. The anterior end shows typical amoebal movement (*arrowheads*) whereas the posterior end still bears a flagellum. See supplementary material V1 for a time-lapse movie. **g**–**h** Amoebal stage after retraction or shedding of the flagellum. Lipid globules in **h** are stained in Nile red. *Scale bars* correspond to 5 μm. *Thick arrows *indicate the flagellum; *thin arrows *indicate the direction of movement

**Fig. 3:**
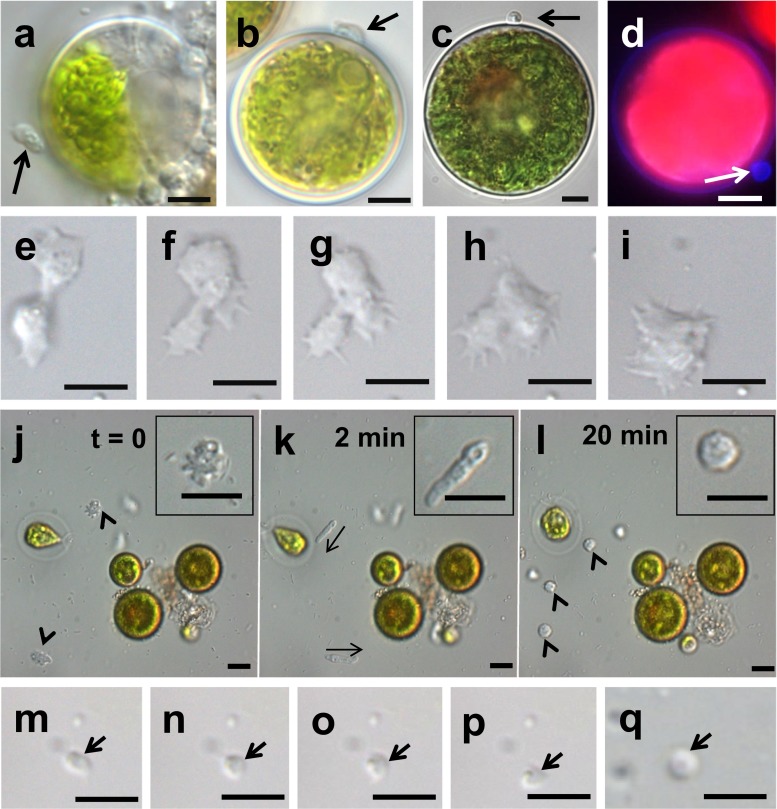
Different amoebal types in *P. sedebokerense*: **a**–**d** Vegetative infectious amoebae that encyst at the surface of a host *Haematococcus* cell (**c**). Calcofluor white staining of the encysted pathogen structure at the algal surface (**d**), *arrows*. **e**–**i** Fusion event between two amoebae. See supplementary material V2 for a time-lapse movie, *arrows*. **j**–**l** Radial amoebae (**j**, *arrowheads*) transitioning into a highly mobile elongate form (**j**, direction of movement indicated by *arrows*), probably upon exposure to high light, and finally encysting (**k**), *arrowheads*. See supplementary material V3 for a time-lapse movie. **m**–**p** Time-lapse pictures of a minute amoeba (*arrow*), taken 60 s apart. **q** Spherical structure, assumed to be an encysted minute amoeba (*arrow*), observed in the infectious flow-through of a diseased *Haematococcus* culture medium filtered on a 1-μm filter. *Scale bars* correspond to 5 μm

Microscopy observation of our *Paraphysoderma*-infected *Haematococcus* cultures confirmed the co-existence of a vegetative and a resting phase of the life cycle as originally described by Hoffman and colleagues (Fig. 1). Sequencing of the 18S rRNA marker of our strain PS1 (GenBank accession KT270356) showed 100 % sequence identity of our isolate with the other two publicly available *P. sedebokerense* sequences (KJ563218, EF565163). In addition, we repeatedly observed fast-moving zoospores in young, 1 to 2-week-old cultures. These zoospores are approximately 3 μm in diameter, spherical in shape and bear one posterior whiplash flagellum (Fig. 2a, b). Swimming movement occurred in a straight line with occasional stops and change of direction as described from several *Physoderma* species (e.g. Sparrow 1964). Moreover, they contain a lipid globule which is visualised by Nile red (Fig. 2b). At the beginning of such microscopic observations, we could always observe high numbers of swimming zoospores, but within 20 min, these numbers decreased. The zoospores reduced their movement and settled down at the bottom of the slides, coinciding with the following transformation (Fig. 2c–f): the previously spherical zoospore adopted an oval shape (Fig. 2c). The end distal to the flagellum formed pseudopodia and developed a typical amoeboid movement, whereas flagella still retained their typical movement (Fig. 2d–f and Suppl. material V1). The retraction or shedding of the flagellum was not directly observed despite repeated attempts, but 1–2 h after starting an observation, zoospores were replaced by large numbers of amoebae. Those amoebae move in a directional fashion: the anterior end forms pseudopodia and appears clear (Fig. 2g) comparable to the zoospore–amoeba transition stage. The posterior end of the amoebae contains lipid globules (Fig. 2h). In several instances, those amoebae were seen on the surface of the algal host *H. pluvialis*. As already described by Hoffman et al. (2008), the amoebae crawled actively over the host’s surface before eventually encysting (Fig. 3a–d), which starts a new cycle of infection. The flagellated zoospore life stage could also be observed at the host surface, but unlike the amoebal stage, the zoospores remained there only for a very short period of time before swimming off again, suggesting that only the amoebal stage is infectious. The release of those zoospores from sporangia was never observed nor the release of amoeboid swarmer cells as previously described (Hoffman et al. 2008). Nonetheless, our observations of a flagellate life stage in *P. sedebokerense* strongly resemble its sister genus *Physoderma* in which uniflagellated zoospores with amoebal behaviour have repeatedly been documented (e.g. Sparrow 1977). It is also worth noting that the presence of amoeboid stages is not uncommon in the *Blastocladiomycota* as infective amoeboid propagules are commonly observed in some species of the genus *Catenaria* (Gleason and Lilje 2009).

In one instance, fusion between two amoebae was observed in liquid algal culture (Fig. 3e–i and Suppl. material V2), whereas in other instances, amoebae approached each other, spent 1–2 min in close contact, and then separated again. Likewise, Hoffman et al. (2008) have described possible conjugation events between amoebae on agar under stressful conditions, and assumed this might lead to the formation of resting sporangia. By analogy with other blastocladiales, which all alternate between a haploid gametophytic (so-called epibiotic) and a diploid sporophytic (so-called endobiotic) phase, we hypothesise that our observation of a fusion between two amoebae could correspond to fertilisation between two gametes. Indeed, detailed and conclusive evidence of fertilisation between epibiotic amoeboid zoospores has been obtained in *Physoderma pulposum* (now called *Urophlyctis pulposa*, Lingappa 1959). Though their observations were slightly less thorough, similar fusion events have been reported by Sparrow and colleagues in many different *Physoderma* species (Sparrow 1975 and refs. therein), leading to the widely held consensus that the life cycle is conserved across the genus. As for *Physoderma* (Lingappa 1959), sexuality seems optional in *P. sedebokerense* with infectious epibiotic zoospores being unfused gametes able to form new epibiotic sporangia parthenogenetically (Fig. 1b).

Furthermore, we were able to observe a second type of amoebae in older (possibly stressed) infected *Haematococcus* cultures (Fig. 3j, k and Suppl. material V3). These amoebae are about 5 μm in diameter, which corresponds to the size of the fused amoebae described above (Fig. 3e, f). They are rather immobile and emit and retract pseudopods in a radially symmetric fashion (Fig. 3j). A few minutes after the beginning of an observation however, they become very elongate and begin to move very rapidly on a straight line parallel to their longest axis (Fig. 3k). We assume that this striking behavioural and morphological transition is triggered by the high light levels necessary for microscopy observation, though this has yet to be formally tested. Notably, phototaxis of zoospores has previously been reported from two other blastocladiales, *Allomyces reticulatus* and *Blastocladiella emersonii* (Saranak and Foster 1997; Avelar Gabriela et al. 2014). Contrary to the amoebae described above, these elongate amoebae do not emit any pseudopod (Fig. 3k) and encyst after roughly 20 min at 20 °C (Fig. 3l). Finally, another type of minute amoebae (∼1.5 μm in diameter, Fig. 3m–p) was found in a few occasions. The presence of such minute amoebae might explain why a filtrate obtained from a diseased *Haematococcus* culture on a pore size as low as 1 μm remains infectious. These very small amoebae might correlate with spherical, 1-μm structures (Fig. 3q) observed in the filtrate, which resembled a miniature form of the encysted amoebae described above. The origin of those different amoebae (radially symmetric and minute) remains to be determined. No attempt has been made to locate meiosis in *P. sedebokerense*. However, ultrastructural studies in other blastocladiales, including *Physoderma maydis* (Lange and Olson 1980), have shown that meiosis occurs within or during the germination of the resting sporangium and leads back to the haploid epibiotic part of the life cycle (Fig. 1k). At this stage, we also cannot rule out the possibility of asexual reproduction (Fig. 1h, m), as known in other blastocladiales such as *Allomyces*. Under this hypothesis, *P. sedebokerense* would undergo parthenogenetic development both in the vegetative (haploid) and resting (diploid) phases, with the transitions between both cycles corresponding to fertilisation (Fig. 1f) and meiosis (Fig. 1i, n) events, respectively. The signals which trigger the transition between the two cycles are currently unknown.

Overall, we propose an updated life cycle that is coherent with earlier observations of *P. sedebokerense* and integrates available knowledge on sexuality in other blastocladiales. Notably, flagellated zoospores can ensure long-range dispersal of the *P. sedebokerense* during an epidemic outbreak. It should be stressed however that the development of *P. sedebokerense* is complex and some important transitions have yet to be directly observed. Additionally, the switch towards and from the vegetative cycle seems facultative, and both vegetative and resting cycles may co-occur in culture depending on the conditions. The timing and nature of the signals that govern zoospore and amoebal release, fertilisation and the switch between the two possible developmental paths remain to be determined. The understanding of this complex life cycle is crucial in order to enable the development of targeted disease management.

## Electronic supplementary material

Below is the link to the electronic supplementary material.

Supplementary material V1 Time-lapse video of zoospore-amoebae transition. In the beginning, in the right part of the video an amoeba can be seen which moves away from the field of observation. Later on fast-swimming zoospores can be observed crossing the image. Video was taken over a 10 min period (speed: 2 frames per sec) (AVI 322,709 kb)

Supplementary material V2 Time-lapse video of amoebal fusion (speed: 1 frame per sec). (AVI 58,324 kb)

Supplementary material V3 Time-lapse video of amoebal movement of one type of amoebae. The amoebae show a very elongated morphology and directional movement. Video was taken over a 15 min period (speed 25 frames per sec) (WMV 3325 kb)

## References

[CR1] Avelar Gabriela M, Schumacher Robert I, Zaini Paulo A, Leonard G, Richards Thomas A, Gomes Suely L (2014). A rhodopsin-guanylyl cyclase gene fusion functions in visual perception in a fungus. Curr Biol.

[CR2] Boussiba S (2000). Carotenogenesis in the green alga *Haematococcus pluvialis*: cellular physiology and stress response. Physiol Plant.

[CR3] Carney LT, Lane TW (2014). Parasites in algae mass culture. Front Microbiol.

[CR4] Carney LT, Reinsch SS, Lane PD, Solberg OD, Jansen LS, Williams KP, Trent JD, Lane TW (2014). Microbiome analysis of a microalgal mass culture growing in municipal wastewater in a prototype OMEGA photobioreactor. Algal Res.

[CR5] CCAP (2015) The Culture Collection of Algae and Protozoa. Medium recipe for 3N-BBM + V (Bold Basal Medium with 3-fold Nitrogen and Vitamins; modified). http://www.ccap.ac.uk/media/documents/3N_BBM_V.pdf. Accessed 29 Aug 2015

[CR6] Chen Z, Lei X, Zhang B, Yang L, Zhang H, Zhang J, Li Y, Zheng W, Tian Y, Liu J, Zheng T (2014). First report of *Pseudobodo* sp, a new pathogen for a potential energy-producing algae: *Chlorella vulgaris* cultures. PLoS ONE.

[CR7] Droop MR (1955). Carotegenesis in *Haematococcus pluvialis*. Nature.

[CR8] Gachon CMM, Strittmatter M, Müller DG, Kleinteich J, Küpper FC (2009). Detection of differential host susceptibility to the marine oomycete pathogen *Eurychasma dicksonii* by real-time PCR: not all algae are equal. Appl Environ Microbiol.

[CR9] Gleason FH, Lilje O (2009). Structure and function of fungal zoospores: ecological implications. Fungal Ecol.

[CR10] Greenspan P, Mayer EP, Fowler SD (1985). Nile red: a selective fluorescent stain for intracellular lipid droplets. J Cell Biol.

[CR11] Hoffman Y, Aflalo C, Zarka A, Gutman J, James TY, Boussiba S (2008). Isolation and characterization of a novel chytrid species (phylum Blastocladiomycota), parasitic on the green alga *Haematococcus*. Mycol Res.

[CR12] James TY, Hoffman Y, Zarka A, Boussiba S (2012). Paraphysoderma sedebokerense, gen. et sp. nov., an aplanosporic relative of Physoderma (Blastocladiomycota). Mycotaxon.

[CR13] Lange L, Olson LW (1980) Germination of the resting sporangia of *Physoderma maydis*, the causal agent of Physoderma disease of maize. Protoplasma 102:323–342

[CR14] Letcher PM, Lopez S, Schmieder R, Lee PA, Behnke C, Powell MJ, McBride RC (2013). Characterization of *Amoeboaphelidium protococcarum*, an algal parasite new to the cryptomycota isolated from an outdoor algal pond used for the production of biofuel. PLoS ONE.

[CR15] Lingappa Y (1959). Sexuality in *Physoderma pulposum* Wallroth. Mycologia.

[CR16] Lorenz RT, Cysewski GR (2000). Commercial potential for *Haematococcus* microalgae as a natural source of astaxanthin. Trends Biotechnol.

[CR17] McBride R, Behnke C, Botsch K, Heaps N, Meenach C (2013) Use of fungicides in liquid systems. PCT Patent Application WO2013056166 A1

[CR18] Porter TM, Martin W, James TY, Longcore JE, Gleason FH, Adler PH, Letcher PM, Vilgalys R (2011). Molecular phylogeny of the Blastocladiomycota (Fungi) based on nuclear ribosomal DNA. Fungal Biol.

[CR19] Saranak J, Foster KW (1997). Rhodopsin guides fungal phototaxis. Nature.

[CR20] Shin W, Boo SM, Longcore JE (2001). Entophlyctis apiculata, a chytrid parasite of *Chlamydomonas* sp. (Chlorophyceae). Can J Bot.

[CR21] Sparrow FK (1964). Observations on chytridiaceous parasites of phanerogams. Arch Mikrobiol.

[CR22] Sparrow FK (1975). Observations on chytridiaceous parasites of phanerogams. XXIII. Notes on Physoderma. Mycologia.

[CR23] Sparrow FK (1977). Observations on chytridiaceous parasites of phanerogams. XXVI. *Physoderma gerhardti* Schroeter on *Phalaris arundinacea* L. Arch Microbiol.

[CR24] Vandenkoornhuyse P, Leyval C (1998). SSU rDNA sequencing and PCR-fingerprinting reveal genetic variation within *Glomus mosseae*. Mycologia.

[CR25] Zhang J, Luo Z, Li J, Liu Z (2013) Production method and device for preventing and treating contamination of *Paraphysoderma sedebokerensis* in *Haematococcus pluvialis*. PCT Patent Application WO2013127280 A1

